# Allograft hemorrhage as a manifestation of carbapenem-resistant *Klebsiella pneumonia* infection in kidney transplant recipients: Case series

**DOI:** 10.1097/MD.0000000000018982

**Published:** 2020-03-27

**Authors:** Zhen Wang, Yeyong Qian, Hongwei Bai, Jintao Yang, Xiang Li

**Affiliations:** Institute of Organ Transplant, The 8th Medical Center of Chinese People's Liberation Army General Hospital, Beijing, 100091, China.

**Keywords:** allograft hemorrhage, carbapenem resistance, kidney transplantation, *Klebsiella pneumonia* infection

## Abstract

**Rationale:**

Carbapenem-resistant *Klebsiella pneumonia* (CRKP) infections have been a concerning threat, especially in organ transplant patients with very high mortality. Allograft hemorrhage associated with CRKP infection has never been described.

**Patient concerns:**

A total of 6 recipients tested positive for CRKP were identified in 297 adult kidney transplant recipients who received kidney from donors according to Chinese type donation after cardiac death (DCD) at our center between January 2006 and December 2017.

**Diagnoses:**

CRKP identification was performed via Vitek 2 system, and the susceptibility was tested by broth microdilution and disk diffusion. Based on the signs of infection and the positive culture, the diagnosis of CRKP infection was established.

**Interventions:**

Therapy with antibiotic such as including ceftazidime-avibactam or tigecycline and surgical control of primary infection source including allograft nephrectomy and/or thorough debridement was administrated.

**Outcomes:**

The most striking aspect was that spontaneous recurrent hemorrhage occurred in all the 6 patients. The mortality of CRKP infection in our study was 50%.

**Lessons:**

CRKP infection possibly due to donor-to-recipient transmission in DCD kidney transplants was essentially a necrotic hemorrhagic inflammation and characterized by recurrent hemorrhage and high mortality. The pre-donation screening for CRKP colonization should be mandatory and, if positive, donation should be contraindicated. And, the effective infection source control such as allograft nephrectomy and/or thorough debridement was important to improve outcomes. Further investigation will be required to further characterize the clinical efficacy of new pharmacotherapeutic schemes including ceftazidime-avibactam.

## 1 Introduction

The recent worldwide outbreaks of multidrug-resistant (MDR) bacteria infections have been a concerning threat, especially in immunocompromised patients.^[1,2]^ Carbapenem-resistant *Klebsiella pneumonia* (CRKP) was identified in 2001,^[3]^ spread to hospitals and became one of the most important pathogens of MDR bacteria.^[4]^ Recently, the increasing threat of CRKP infection in organ-transplanted patients has been highlighted, due to difficulty in treatment and high mortality.^[5–8]^ Clinical studies have shown that infections with CRKP were consisted of bloodstream infections,^[9]^ urinary tract infections,^[10]^ pneumonia,^[11]^ central venous catheter-associated infection,^[12]^ and surgical site infections.^[13,14]^

Allograft hemorrhage was a severe and potentially lethal complication of renal transplantation. Previous studies reported that allograft infection, such as Mucoraceous^[15]^ and Aspergillus,^[16]^ was a rare cause of allograft renal artery rupture and hemorrhage in kidney recipients. Unlike previous cases of allograft hemorrhage associated with fungal infections, we describe 6 cases of allograft hemorrhage in kidney recipients caused by CRKP infections. According to our knowledge, the allograft hemorrhage due to CRKP has never been described in transplant recipients.

## 2 Case description

The retrospective cohort study included 297 adult (older than 18 years of age) kidney transplant recipients who received kidney from donors according to Chinese type donation after cardiac death (DCD)^[17]^ at our center between January 2006 and December 2017. CRKP identification was performed via Vitek 2 system, and the susceptibility was tested by broth microdilution and disk diffusion. CRKP were confirmed as resistant to carbapenem antibiotic. The pre-donation screening for CRKP colonization in donors and recipients was routinely performed. None of the donors were confirmed to be infected or colonized with CKRP prior to the donation. And, none of the recipients have positive cultures for CRKP around the time of transplant.

A total of 6 recipients tested positive for CRKP were identified, with an incidence of 2.0% (6/297). All the patients’ main clinical–pathological characteristics were described in Tables 1 and 2. Also, the antibiotic susceptibility was summarized in Table 3.

**Table 1:**
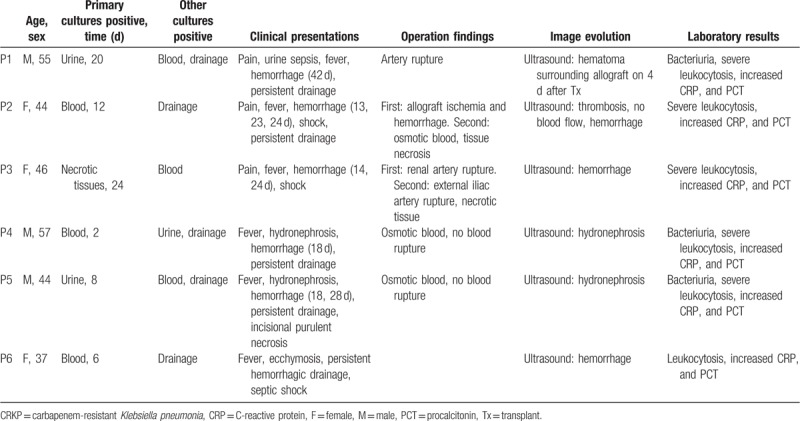
Clinical characteristics of recipients with CRKP infections.

**Table 2:**
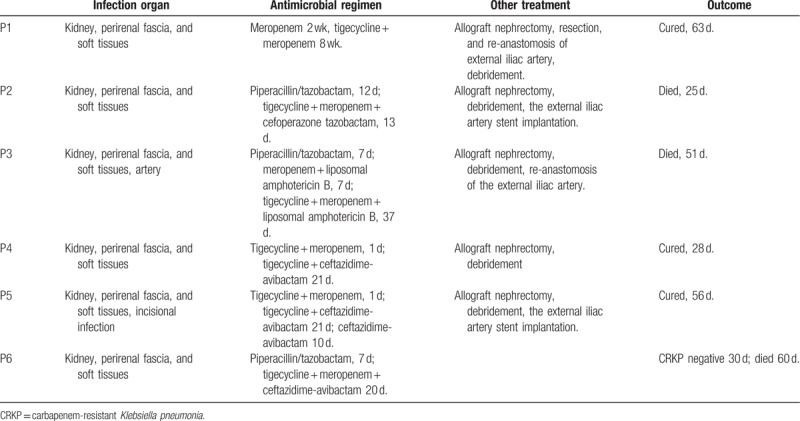
Treatment and outcomes of transplant recipients with CRKP infection.

**Table 3:**
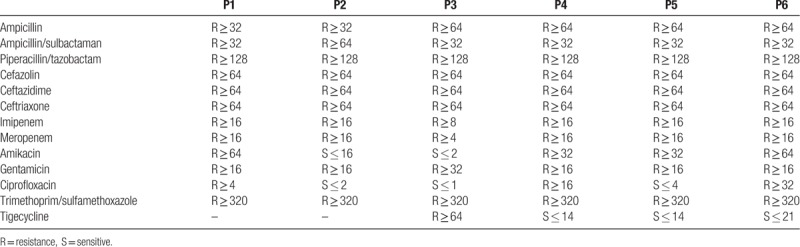
Mean inhibitory concentrations (mg/L) for *Klebsiella pneumonia* isolated from patients.

### 2.1 Case 1

**Figure 1:**
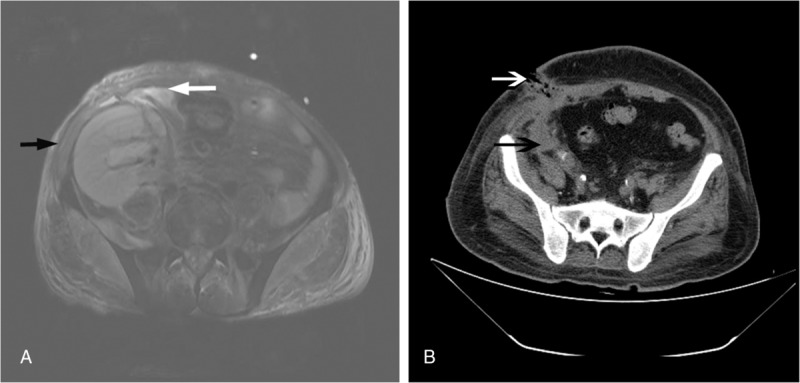
(A) Magnetic resonance imaging from patient 1 showed an enlarged renal graft, abdominal and pelvic soft tissues with stripe and grid high signal (black arrow), and pelvic peritoneal fluid signal (white arrow); which was suggestive of the transplanted kidney and perirenal tissue infection. (B) Computerized tomographic scanning of case 5 showed big low density shape shadow in the iliac fossa (black arrow) and soft tissue defects at the incision (white arrow), indicating massive fluid and incision infection.

**Figure 2:**
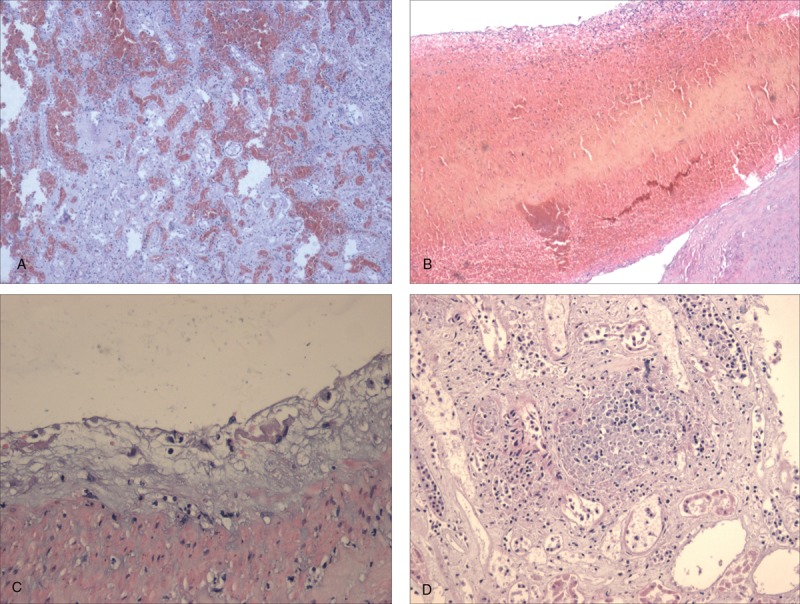
Pathological features of case 1 showed presence of acute inflammatory cell infiltration accompanied by massive hemorrhage and infarction (A); thrombus formation in the renal portal vessels (B); mucoid degeneration of the intima of the arteries (C), and microabscess (D) in the transplanted kidney under the light microscope after hematoxylin–eosin stain.

A 55-year-old man with end-stage renal disease (ERSD) resulting from glomerulonephritis underwent kidney transplant in July 2016. His initial posttransplant course was good with proper allograft function. On posttransplant week 1, the patient had a persistent fever and bacteriuria, associated with a progressively increased level of creatinine (Cr, 2.36 mg/dL) and infectious parameters (C-reactive protein [CRP], 137 mg/L; procalcitonin [PCT], 1.0 ng/mL), was empirically treated with meropenem. Because of the condition deterioration, the patient was transferred to our hospital on posttransplant day (PT) 14. Blood and urine grew CRKP, which was extensively drug resistant and sensitive only to amikacin and tigecycline on PT 20. Thereafter, tigecycline was immediately given. The fever was not relieved, and a severe leukocytosis, anaemia, and leukocyturia were found. Magnetic resonance imaging showed an enlarge kidney with a moderate perirenal hematoma, which was suggestive of the allograft infection (Fig. 1A). On PT 42, the possibility of acute renal hemorrhage is considered due to the pain in the kidney transplantation area and the decrease of blood pressure. The emergency exploration surgery showed that the artery of transplanted kidney was pale, poorly elastic, and severely damaged in the vessel wall. A rupture at the anastomotic stoma of the renal artery and the external iliac artery was found, and allograft nephrectomy and resection and re-anastomosis of external iliac artery were performed. Pathological examination showed acute inflammatory cell infiltration accompanied by massive hemorrhage and infarction; thrombus formation in the renal portal vessels; mucoid degeneration of the intima of the arteries and microabscess in the transplanted kidney (Fig. 2). During the next 2 weeks after nephrectomy, a large number of purulent exudates were drained in the allograft area and CRKP was positive. After 2 weeks of nephrectomy, the drainage was gradually reduced. On PT 63, the blood and drainage fluid culture of CRPK were negative. The patient was recovered and discharged from the hospital.

### 2.2 Case 2

**Figure 3:**
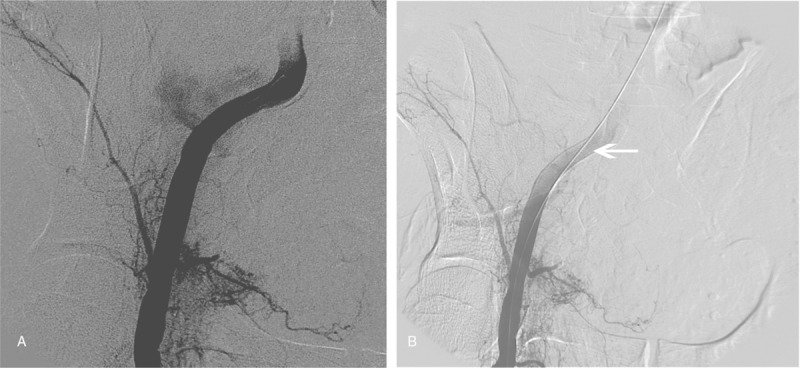
Comparison of angiography images from patient 6 obtained before (A) and after (B) the external iliac artery covered stent implantation (white arrow) to resolve emergency hemostasis. (A) Large hemorrhage from the right external iliac artery. (B) No evidence of hemorrhage.

A 44-year-old woman with ERSD resulting from glomerulonephritis underwent kidney transplant in July 2016. On PT 3, level of Cr was normal. On PT7, the patient had mild fever. Because of the blood CRKP-positive, she was administered tigecycline plus with meropenem. On PT 13, severe pain and hematogenic shock suddenly occurred. The ultrasound examination showed the thrombosis of the renal allograft artery without blood flow and the signs of hemorrhage of allograft. The emergency surgery showed ischemia and necrosis of graft, renal hemorrhage, and no obvious vascular defects. And, nephrectomy was performed. After nephrectomy, a great deal of hemorrhagic purulent liquid that grew CRKP and Bauman Acinetobacter was continuously drained. Therefore, the antibiotics adjusted to cefoperazone tazobactam plus tigecycline. On PT 23 after hemodialysis, the additional hemorrhage was considered because of the durative bloody drainage volume associated with hemodynamic instability. The external iliac artery stent implantation was performed in the emergency digital subtraction angiography (DSA) to stop bleeding (Fig. 3). On the second day, the third hemorrhagic shock occurred again. Multiple tissue hemorrhage characterized with osmotic blood in the iliac fossa was found in the third emergency surgery. She ultimately died on PT 25.

### 2.3 Case 3

A 44-year-old woman with ERSD resulting from glomerulonephritis underwent kidney transplant at our hospital in July 2016. Post-transplantation, the recipient showed good general condition without fever, but higher infectious index (CRP, 110 mg/L; PCT, 1.2 ng/mL). On PT 14, severe pain and hematogenic shock suddenly occurred. An emergency exploratory operation revealed bleeding from a rupture with a diameter of approximately 5 mm in the renal artery. The kidney and fascia surrounding the graft appeared normal. Based on the suspicion for mucormycosis, allograft nephrectomy was performed, immunosuppression was stopped, and liposomal amphotericin B was administered. On PT 23, the second exploratory operation because of another hemorrhagic shock revealed another rupture with a diameter of approximately 2 mm at the proximal end of external iliac artery. An amount of dead muscle was encountered and the peritoneal layer appeared dusky. Surgical debridement and re-anastomosis of the external iliac artery were performed. And, the necrotic abdominal tissues and blood grew CRKP. Acinetobacter baumannii was cultured in the blood on PT30, and the antibiotics were adjusted to cefoperazone tazobactam and tigecycline. Despite intensive drugs, the patient died on PT51.

### 2.4 Case 4

A 37-year-old male patient with ERSD caused by chronic glomerulonephritis underwent renal transplantation in our hospital in May, 2017. On PT 2, the donor's blood cultures grew CRKP only sensitive to tigecycline and compound sulfamethoxazole. Then, tigecycline combined with ceftazidime-avibactam was immediately applied to the patient. On PT3, level of serum creatinine was normal. Within 1 week after transplant, routine urinalysis showed bacteriuria with normal leukocyte. 1 week after transplant, he had a persistent leukocytosis and fevers. Ultrasound due to an elevated Cr showed persistent hydronephrosis. Therefore, percutaneous nephropyelostomy was performed, and a pyelostomy tube was placed. The hemorrhage in puncturing area was found, the blood pressure dropped and the hemorrhagic shock occurred on PT18. Emergency exploration showed severe bleeding in the surrounding tissue of the transplanted kidney and no vascular rupture. So, nephrectomy and thorough debridement were performed. A great deal of hemorrhagic purulent liquid was continuously drained from the transplanted kidney area, and was positive for CRKP in the following week. On the 10th day after nephrectomy, the drainage fluid disappeared. Eventually completing 1 month's tigecycline plus ceftazidime-avibactam therapy, the infection was cured without evidence of CRKP in the culture of urine and blood.

### 2.5 Case 5

A 44-year-old man with ERSD caused by diabetes mellitus and hypertension received the donor kidney from the same donor as the case 4. Because of delayed graft function, hemodialysis was required. On PT2, tigecycline plus with ceftazidime-avibactam was used in the prevention and treatment of CRKP. The same as case 4, allograft hydronephrosis was detected and the percutaneous transplanting renal puncture and fistula drainage was performed on PT8, and CRKP grew in the urine drainage. In the next week, the patient had fever accompanied by a continuous increase of bacteriuria and leukocytosis. On PT 18, bleeding was coincidentally found at the puncture and drainage area. The rupture of the anastomotic stoma of the transplanted renal artery and the external iliac artery was revealed in the emergency exploratory operation, and the graft nephrectomy combined with debridement was performed. On PT28, the recurrence of acute massive hemorrhage occurred after hemodialysis. Therefore, the external iliac artery covered stent was used for emergency hemostasis. A large number of hemorrhagic purulent drainage and incisional infection (Fig. 1B) were found within the next 2 weeks. CRKP were recovered from both the drainage and incisional infection secretions. After 8 weeks of tigecycline plus with ceftazidime-avibactam treatment, the infection was cured without evidence of CRKP.

### 2.6 Case 6

A 37-year-old female patient with uremia caused by chronic glomerulonephritis underwent kidney transplant in our hospital in May, 2017. Hemodialysis was necessary because of delayed graft function. The results of renal perfusion and donor blood bacteria culture were negative. On PT3, the patients suffered fever with the increase of leukocyte and neutrophils ratio. On PT6, the blood culture confirmed CRKP-positive. And, the drug sensitivity test showed that it was sensitive only to tigecycline and sulfamethoxazole. The antibiotics were changed to tigecycline plus meropenem. There was a large area ecchymosis in the recipient's right lower abdomen and lower back. Subsequently, the skin was marked by inflammation and necrosis, and diagnosed as necrotizing fascia tissue inflammation. On PT10, the signs of renal allograft hemorrhage were detected by ultrasonography. In the ensuing days, a great deal of hemorrhagic fluid which culture was positive for CRKP was continuously drained in the transplanted kidney area. However, the results of blood and drainage culture showed that CRPK was negative on PT30. Eventually, the patient continued to deteriorate, suffered from severe septic shock, and died on PT60.

## 3 Discussion

CRKP infections in our study occurred during different time periods, suggesting not relation to infection outbreak. Most of CRKP infections (5/6) developed immediately in the first moth after transplant, were more likely to occur within 2 weeks after transplant. Within the same time period, none of recipients from living donor kidney developed CRKP infection. It was noteworthy that the 2 kidney recipients from the same donor who was confirmed to be colonized with CRKP after donation developed infection. Additionally, the risk of donor-derived infections has also been previously described in deceased-donor renal transplant.^[18]^ Therefore, donor-to-recipient transmission (probably via allograft) was considered to be the most likely cause of the infection. Moreover, it was assumed that donors were at high risk for acquiring multidrug-resistant pathogens including CRKP infection because of poor functional debilitated status, prolonged intensive care units admissions, mechanical ventilation, more exposure to multiple antimicrobials that may fostered the selection of resistant strains.^[19]^ Given the risk of transmission of infection from donor, pre-donation screening for CRKP colonization in donors is of paramount importance for the prevention of subsequent infections in transplant.^[20,21]^ In times of delay the currently culture-based techniques, screening performed on the day of donation cannot be taken into account the bacteremia carrier status.^[22]^ Therefore, the clinical risk stratification strategy in conjunction with systematic screening may be helpful in identifying risk of donor bacteria carriage and prevention of donor-derived infections.

The most common clinical presentation of our case series included systemic inflammatory response syndrome (SIRS) associated with infection, septic shock, and recurrent hemorrhage. Intraoperative findings revealed ruptures in the renal artery or iliac artery, dead fascia, and muscle. Post-nephrectomy, a large amount of purulent bloody fluid was persistently obtained from the drainage tube in the iliac fossa. All cultures (blood, urine, drainage, subcutaneous fluid) in the 6 aforementioned cases were positive for CRKP. In addition, CRKP also grew in the perirenal dead tissue obtained from nephrectomy and biopsy specimens. Pathological examinations confirmed the presence of diffuse hemorrhagic necrotic lesions with lymphocyte infiltration, vascular wall inflammation cells erosion (vasculitis), and thrombosis in the blood vessels. In times of clinical manifestations, pathological findings and evidence of positive microbiological finding, we firstly elucidated that CRKP infection was essentially a necrotic hemorrhagic inflammation. This was line with results from previous studies in which the necrotizing inflammation associated with CRKP including abscesses and necrotizing soft tissue infection developed in organ recipients.^[13,14]^ The necrotizing fasciitis associated with CRKP was also reported in a heart transplant case.^[23]^ Furthermore, another case showed that CRKP pyelonephritis caused perirenal hematoma.^[24]^

The most striking aspect was that recurrent hemorrhage occurred in all the 6 recipients, suggesting that the anastomotic dehiscence and arterial rupture were associated with CRKP infection. CRKP infections had different clinical presentations due to the anatomic location of infection, was characterized by strong local invasion and low probability of distant organ proliferation. When the infection only involved in the allograft within about 1 week, the patients presented with fever, urinary tract infections, elevated white blood cells, or asymptomatic symptoms. With the massive bacterial replication, CRKP rapidly spread from kidney to the contiguous tissue by direct inoculation within 2 to 3 weeks after transplantation. Eventually, tissues around allograft including deep fascia, subcutaneous tissue, and arteries were infected by CRKP. Subsequently, this resulted in multiple necrotic inflammatory lesions, multiple abscess cavities filled purulent fluid, and persistent drainage. Mechanical destruction of vessel wall by infiltration of inflammatory cells and endothelial cell contributed to spontaneous arteries rupture, sudden hemorrhage, and hemorrhagic shock. To our knowledge, this was the first reported cause of spontaneous recurrent allograft hemorrhage in kidney recipients due to CRKP infection. In order to avoid the serious consequences of later vascular complications, the anastomosis of the donor renal artery to the internal iliac artery should be preferred when the donor was suspected to be infected. Optimization of anticoagulants might reduce the risk of hemorrhage during hemodialysis in CRKP infected recipients.

The mortality of CRKP infection in our study was 50%, and almost of the deaths occurred within 4 weeks after the infection. Previous studies also demonstrated that the total CRKP infection mortality rate of the transplant recipients was as high as 40% to 75%,^[5,6]^ and the 30-days mortality rate was 50% to 60%.^[7,8]^ CRKP was resistant to most of the antibiotics, but sensitive only to polymyxin and tigecycline,^[2,5]^ which was the main cause of high lethality. We found that CRKP was only partly sensitive to tigecycline (sensitivity 50%). Early diagnosis of CRKP infection was difficult, and sensitive antibiotic treatment was often delayed,^[4]^ which was another important factor leading to high mortality. The accuracy rate of early application of antibiotics in accordance with the drug sensitive results was only 50% in our study, which was line with the previous reported result that the early treatment rate of sensitive antibiotics was only 35% in CRKP infection.^[25]^ Moreover, the toxic side effects of antibiotics, the immunocompromised state, graft insufficiency required hemodialysis, and concurrent mixed infections were also important causes of high mortality. Previous clinical trials reported that allograft resection improved the success rate of infection cure.^[11,26]^ Moreover, the study of liver transplant recipients showed that completely debridement was essential to CRKP necrotizing soft tissue infections.^[14]^ All the 3 surviving recipients in our study underwent allograft nephrectomy, thorough debridement, and drainage. In addition, no distant infection in the lungs, liver, and brain was found in our study. At the same time, Simkins et al also reported 13 cases of CRKP infection without the diffusion of distant organs.^[26]^ The most possible likely explanation was that the patient had died before the infection of the distant organs. Therefore, the effective infection source control was great significance to improve the survival rate in recipients with CRKP infection.

The clinical efficacy of ceftazidime-avibactam against CRKP was corroborated by our observations. The resistance rate of CRKP to polymyxin and tigecycline was increasing.^[27,28]^ The research in New York confirmed that the resistance rate of CRKP to polymyxin was 9% to 27%.^[29]^ And, the existence of CRKP resistant to tigecycline was also found in the study of Greece.^[28]^ Two survived patients in our series survived were treated with ceftazidime-avibactam in conjunction with surgical source control, indicating that ceftazidime-avibactam represented a great potential agent for the treatment of CRKP. Recent clinical trials have proved that the CRKP infection in immunocompromised hosts was successfully treated with a ceftazidime-avibactam-based therapy.^[30]^ However, more clinical data are available to better evaluate the clinical efficacy.^[31]^

Some limitations of our study should be noted. First, this study was only a retrospective single center study including a small number of infections, and may have introduced bias. Second, the culture method to confirm CRKP infection in our study was less sensitive than molecular biology and may lead to false negatives. The lack of gene identification of strains and the mechanism of drug resistance in our study were the third shortcoming.

To summarize, CRKP infection possibly due to donor-to-recipient transmission in DCD kidney transplants was essentially a necrotic hemorrhagic inflammation and characterized by recurrent hemorrhage and high mortality. These data provided that pre-donation screening for CRKP colonization should be mandatory and, if positive, donation should be contraindicated. Furthermore, the effective infection source control such as allograft nephrectomy and/or thorough debridement was important to improve outcomes. Nevertheless, further investigation will be required to explore new pharmacotherapeutic scheme including ceftazidime-avibactam.

## Author contributions

Zhen wang, Yeyong Qian, Hongwei Bai, Xiang Li, and Jintao Yang performed transplants.

Zhen wang, Xiang Li, and Jintao Yang collected, analyzed data, and interpreted results.

Zhen Wang prepared figures, drafted manuscript, and edited manuscript.

**Conceptualization:** Zhen Wang.

**Data curation:** Zhen Wang, Yeyong Qian, Xiang Li, and Jintao Yang.

**Formal analysis:** Zhen Wang.

**Investigation:** Zhen Wang.

**Methodology:** Zhen Wang, Xiang Li.

**Resources:** Zhen wang, Yeyong Qian, Hongwei Bai, Xiang Li, Jintao Yang.

**Writing – original draft:** Zhen Wang.

**Writing – review & editing:** Zhen Wang.
